# Correction: Indoor methane consistently above outdoor levels in homes with natural gas service

**DOI:** 10.1371/journal.pone.0357112

**Published:** 2026-08-26

**Authors:** Nathan G. Phillips, Robert Ackley, Andee Krasner

There is an error in affiliation 3 for author Andee Krasner. The correct affiliation 3 is: Independent Public Health Consultant, Boston, Massachusetts, United States of America.

In Table 1, all values in Column 6 entitled “Standard Error” are miscalculated. Please see the correct Table 1 here.

In S1 Appendix, most values in Column 8 of Table 4, entitled "“Third Floor Minus Outdoor Gas,” are incorrect. It can be viewed below.

**Table 1:** Summary statistics of [CH_4_] concentrations in parts per million (ppm) outdoors and indoors. Indoor concentrations are unadjusted for outdoor concentrations. P-values are for differences between indoor and outdoor [CH_4_].

Category	Outdoor/ Indoor Floor	Mean (ppm)	Standard Deviation	Number of homes (n)	Standard Error	Median (ppm)	P-value
Gas	Outdoors	2.04	0.10	175	0.01	2.02	
No Gas		2.03	0.09	20	0.02	2.01	
Gas	Basement	3.95	3.80	164	0.30	2.83	< 0.0001
No Gas		2.06	0.27	18	0.06	2.02	>0.10
Gas	First Floor	3.35	2.31	166*	0.18	2.63	< 0.0001
No Gas		2.13	0.25	20	0.06	2.07	0.04
Gas	2nd Floor	3.33	2.18	141	0.18	2.65	< 0.0001
No Gas		2.11	0.16	12	0.05	2.08	>.10
Gas	3rd floor	3.32	2.77	41	0.43	2.50	0.005
No Gas		2.10	0.21	4	0.10	2.03	**

*Note that the first floor of a dwelling unit may not be the first floor of a building, for example in multi-story apartments.

**P-value is not presented due to the small number of homes with 3rd floors in the no gas group.

## Supporting information

S1 Appendix Instrument calibration check. We tested the analyzer prior to the beginning of the survey, on February 29, 2024; during a midpoint of the survey, on April 23, 2024, and near the conclusion of the survey on June 19, 2024, against nominal 0.0 ppm; 2.0 ppm and 10 ppm test gasses in ultrapure air. The test gas tanks (Scott-Marrin, Riverside, CA USA) were certified to contain < 0.01 ppm; 2.072 ppm; and 10.32 ppm [CH4] respectively (+/- 1% NIST). Test gasses were supplied to the analyzer using Tedlar bags filled with test gasses and connected to the analyzer inlet during normal operation. Tedlar bags of test gas supply were maintained until the graphical data display indicated the analyzer [CH4] reading stabilized near the nominal value. Table 3 shows the match between analyzer values of [CH4] and test gas values. These results demonstrate that our analyzer was working properly and with adequate precision for the study.
(DOCX)
