# Supplementary material for: Indoor methane consistently above outdoor levels in homes with natural gas service
Source: PLoS One. 2026 May 19;21(5):e0348496. doi: 10.1371/journal.pone.0348496 (PMC13186372; doi:10.1371/journal.pone.0348496)
Supplement: Attachment — Submitted filename: Response to Reviewers_revised.pdf [file pone.0348496.s002.pdf]

Boston University

Department of Earth and Environment
685 Commonwealth Avenue
Boston, Massachusetts 02215–1401

I. Responses to Journal Requirements

1. Please ensure that your manuscript meets PLOS ONE's style requirements, including those for file naming. The PLOS ONE style templates can be found at
https://journals.plos.org/plosone/s/file?id=wjVg/PLOSOne_formatting_sample_main_body.pdf and
https://journals.plos.org/plosone/s/file?id=ba62/PLOSOne_formatting_sample_title_authors_affiliations.pdf

2. Thank you for stating the following in the Acknowledgments Section of your manuscript:

“Funding for this study was provided by ZeroCarbonMA.”

We note that you have provided funding information that is not currently declared in your Funding Statement. However, funding information should not appear in the Acknowledgments section or other areas of your manuscript. We will only publish funding information present in the Funding Statement section of the online submission form.

Please remove any funding-related text from the manuscript and let us know how you would like to update your Funding Statement. Currently, your Funding Statement reads as follows:
“This research was funded by Zero Carbon Massachusetts.”

We have removed this statement from the acknowledgments and confirm that it is in the Funding Statement section of the online submission form. Co-authors Nathan Phillips, Robert Ackley, and Andee Krasner were funded by Zero Carbon Massachusetts and we have included funding statements for the authors.

Please include your amended statements within your cover letter; we will change the online submission form on your behalf.

3. Thank you for stating the following financial disclosure:

“This research was funded by Zero Carbon Massachusetts.”
Please state what role the funders took in the study. If the funders had no role, please state: "The funders had no role in study design, data collection and analysis, decision to publish, or preparation of the manuscript."
If this statement is not correct you must amend it as needed.
Please include this amended Role of Funder statement in your cover letter; we will change the online submission form on your behalf.

We have explicitly addressed this requirement in the cover page of this cover letter, above.

4. Thank you for stating the following in the Competing Interests section:

“The authors have declared that no competing interests exist.”
We note that one or more of the authors are employed by a commercial company: “Gas Safety, Inc.”
1. Please provide an amended Funding Statement declaring this commercial affiliation, as well as a statement regarding the Role of Funders in your study. If the funding organization did not play a role in the study design, data collection and analysis, decision to publish, or preparation of the manuscript and only provided financial support in the form of authors' salaries and/or research materials, please review your

statements relating to the author contributions, and ensure you have specifically and accurately indicated the role(s) that these authors had in your study. You can update author roles in the Author Contributions section of the online submission form.

We have explicitly included the role of Gas Safety, Inc. in this research in the funding statement in the cover page of this response letter, above. We also updated the Author Contributions section of the online submission form to be consistent with this statement.

Please also include the following statement within your amended Funding Statement.
"The funder provided support in the form of salaries for authors [insert relevant initials], but did not have any additional role in the study design, data collection and analysis, decision to publish, or preparation of the manuscript. The specific roles of these authors are articulated in the 'author contributions' section."
If your commercial affiliation did play a role in your study, please state and explain this role within your updated Funding Statement.

Our funding statement above is responsive to these requirements.

2. Please also provide an updated Competing Interests Statement declaring this commercial affiliation along with any other relevant declarations relating to employment, consultancy, patents, products in development, or marketed products, etc.
Within your Competing Interests Statement, please confirm that this commercial affiliation does not alter your adherence to all PLOS ONE policies on sharing data and materials by including the following statement: "This does not alter our adherence to PLOS ONE policies on sharing data and materials." (as detailed online in our guide for authors http://journals.plos.org/plosone/s/competing-interests) . If this adherence statement is not accurate and there are restrictions on sharing of data and/or materials, please state these. Please note that we cannot proceed with consideration of your article until this information has been declared.
Please include both an updated Funding Statement and Competing Interests Statement in your cover letter. We will change the online submission form on your behalf.

These two statements are included as bolded text in the cover page of this letter.

5. We note that Figure 1 in your submission contain map images which may be copyrighted. All PLOS content is published under the Creative Commons Attribution License (CC BY 4.0), which means that the manuscript, images, and Supporting Information files will be freely available online, and any third party is permitted to access, download, copy, distribute, and use these materials in any way, even commercially, with proper attribution. For these reasons, we cannot publish previously copyrighted maps or satellite images created using proprietary data, such as Google software (Google Maps, Street View, and Earth). For more information, see our copyright guidelines: http://journals.plos.org/plosone/s/licenses-and-copyright.

We require you to either (1) present written permission from the copyright holder to publish these figures specifically under the CC BY 4.0 license, or (2) remove the figures from your submission:

We decided that Figure 1 was unnecessary since all of the information therein is available in our supplementary data document. Therefore, this figure has been removed and the original Figure 2 is now Figure 1 in the revision, and so on.

6. Please upload a copy of Figure 4, to which you refer in your text on page 11. If the figure is no longer to be included as part of the submission please remove all reference to it within the text.

Thank you for catching this error, which was a misnumbered Figure. It is now properly referenced as Figure 2.

7. We note that there is identifying data in the Supporting Information file "PhillipsAckleyKrasner2025SupportingInformation.xlsx". Due to the inclusion of these potentially identifying data, we have removed this file from your file inventory. Prior to sharing human research participant data, authors should consult with an ethics committee to ensure data are shared in accordance with participant consent and all applicable local laws.
Data sharing should never compromise participant privacy. It is therefore not appropriate to publicly share personally identifiable data on human research participants. The following are examples of data that should not be shared:
-Name, initials, physical address
-Ages more specific than whole numbers
-Internet protocol (IP) address
-Specific dates (birth dates, death dates, examination dates, etc.)
-Contact information such as phone number or email address
-Location data
-ID numbers that seem specific (long numbers, include initials, titled "Hospital ID") rather than random (small numbers in numerical order)
Data that are not directly identifying may also be inappropriate to share, as in combination they can become identifying. For example, data collected from a small group of participants, vulnerable populations, or private groups should not be shared if they involve indirect identifiers (such as sex, ethnicity, location, etc.) that may risk the identification of study participants.
Additional guidance on preparing raw data for publication can be found in our Data Policy (https://journals.plos.org/plosone/s/data-availability#loc-human-research-participant-data-and-other-sensitive-data) and in the following article: http://www.bmj.com/content/340/bmj.c181.long.

Please remove or anonymize all personal information "Address", ensure that the data shared are in accordance with participant consent, and re-upload a fully anonymized data set. Please note that spreadsheet columns with personal information must be removed and not hidden as all hidden columns will appear in the published file.

After reviewing our Supplementary Data file and the helpful guidelines above, we believe that the only data that should not have been shared are the dates and times that sampling was performed. We feel that street names (without address numbers) are sufficiently anonymized yet provide a reasonable level of spatial granularity commensurate with the spatial variability in gas distribution service and pipeline infrastructure. Based on this consideration, we have removed dates and times of sampling, and have otherwise left the supplementary data in place in our revised submission.

8. If the reviewer comments include a recommendation to cite specific previously published works, please review and evaluate these publications to determine whether they are relevant and should be cited. There is no requirement to cite these works unless the editor has indicated otherwise.

Reviewers did not recommend citing additional published works.

II. Responses to Reviewer Comments

Reviewer #1: The paper is very well written and I only have a few minor comments to address:

"relatively large gas leaks that 116 unnecessarily add to ratepayer bills and to climate warming may produce smaller elevations of 117 [CH4] due to poor home insulation and high air exchange rates" Smaller than what? It's unclear what this is referencing.

We clarified this sentence on lines 115-118 (revised track changes version) to "relatively large gas leaks that unnecessarily add to ratepayer bills and to climate warming may produce relatively smaller elevations of [CH_4] in homes with poor insulation and high air exchange rates, compared to the same sized leak in better insulated and/or less well ventilated homes".

Line 144: How long were residents instructed to keep their windows and doors closed before the visit? How long would be required to ensure the measurement is reflective of worst-case scenario?

We clarified (lines 146-147 revised track changes version) this sentence by adding the clause below in italics: "Participants were asked to keep doors and windows closed prior to the visit, *typically a day prior to visits*, but normal traffic in and out of the homes was not curtailed." This addition is consistent with text in materials and methods which states "This work was completed during late winter to late spring 2024, when New England homes typically keep windows and exterior-facing doors closed. To ensure comparable indoor conditions, householders were asked to minimize ventilation through windows or doors for at least a day prior to measurements. We did not request any further special weather sealing or insulation."

Line 138: For the data collected in the warmer months, I'm not sure the heating being 'on' would have much an impact. Even though it might be 'on' on the thermostat it's unlikely there would be calls for heating how was this addressed?

The majority of leaks/emissions from heating systems and in general were found at supply line fitting to the appliances. Gas stoves and water heaters had fitting leaks inside when off. So in our opinion having the heat on or off made little difference. We have added a paragraph (lines 296-302 in revised marked up version) to address this thoughtful point.

On line 52, it states that measurements were taken at floor level but on line 152, it says measurements were taken in the middle of the floor level. Which was it? Was there any indication of heating system runtime or gas appliance use during the measurement period? Also, how frequently were the measurements taken between 7am-5pm in each home.

Thank you for pointing out this inconsistency. We clarified in the abstract that measurements were taken at mid-floor level (line 51 of revised tracked changes version).

On line 60/61, it says that leaks from gas equipment were confirmed but on line 178, it states this was done when convenient/possible for homeowner.

We clarified (line 60, tracked changes revision) that "In 91% of *a subset of* homes investigated, leaks from gas equipment were confirmed. This subset amounted to 137 of the 175 homes that had gas service, meaning that we confirmed a gas leak in 124 of the 137 homes in which we were able to search for a gas leak. Some pipes were also inaccessible due to concealment by walls and ceilings. This information was included in the text on lines 256-258.

Line 209, what could cause the marginal methane increases in non-gas homes?

We believe it is plausible that these relatively very small [CH4] elevations in homes without gas service may have been caused by biotic factors such as flatulence or improperly functioning plumbing. We have added this consideration to the text (lines 212-213 of tracked changes revision).

—---

Reviewer #2: This manuscript examines a significant and urgent public health and environmental concern: the elevated indoor methane levels in homes utilising natural gas services. This subject pertains to indoor air quality research, climate science, and energy policy. The authors provide empirical measurements obtained from various households, offering valuable evidence of methane accumulation in real-world scenarios.
The study is well-motivated, and the results provide useful information that adds to the growing body of research on indoor emissions from natural gas appliances. There are, however, a few places where the manuscript could use more explanation, more methodological detail, and a stronger discussion of the context. These changes will make the results more scientifically sound and easier to understand.

Major Comments
1. Although the general sampling method is explained, more information is needed to make it possible to repeat the process. Please explain:

• The reasons for choosing the homes that will participate (for example, the age of the building, the type of building, and the way it is ventilated).

On lines 129-132 (tracked changes revision) we state: "Homes in urban and rural areas were selected across eastern Massachusetts and Rhode Island (Figure 1) using a combination of quota sampling [26] and snowball sampling [27]. Quota sampling was used to achieve a relatively balanced set of households of different types and ages (single-family, multi-family, and multi-unit apartments)." We believe that this sentence and the quota sampling approach generally captures our intention to sample from a diverse housing stock in terms of age and type of building, but acknowledge important limitations of our sampling in the Discussion section starting on line 399 (tracked changes revision). Moreover, as important factors as they are, ventilation type or degree and type of insulation was not explicitly considered in this study, as it was beyond our ability to conduct tests for whole-house ventilation rate with tools such as a blower door. We agree that tests for ventilation rate and type would be an important factor to include in future studies, and have added that sentence to the conclusions on line 404 (tracked changes revision).

• If sampling was done during certain times of the year or with controlled ventilation.

On lines 137-138 (tracked changes revision) we describe that sampling was performed during the period from February 24, 2024 to June 20, 2024. We did not use controlled ventilation but generally asked household participants to close windows and doors for typically an hour prior to our arrival for sampling. To otherwise mimic ordinary household conditions, we did not ask participants to refrain from entering or leaving the house through doorways.

• The calibration and detection limits of the methane monitors used.

Lines 157-161 (tracked changes revision) contains the make and model of the analyzer used as well as the lower detection limit, or precision (0.003 ppm) and Appendix 1 contains calibration check data we performed on three occasions spanning the study period.

• If any co-pollutants (like NO_2, CO_2, or VOCs) were measured to put gas use or combustion events in context.

This study was intended only for the simple purpose of measuring elevations in methane in homes serviced with gas. We are aware of and discuss concerns of co-pollutants from uncombusted and combusted natural gas, which we hope provide important broader context for this study.

More information will make people more sure that they can compare samples from inside and outside.

2. The manuscript would benefit from further elucidation regarding:
• The identification and management of outliers.

Lines 235-238 (tracked changes revision) and the figure caption for new Figure 1 (originally Figure 2) discuss "outliers", making it clear that these rare high concentration values are real and a key feature of leaks across the entire natural gas process chain. Therefore, and without any reason to suspect measurement errors, we do not exclude such leaks from this study but rather highlight the importance of recognizing the relatively small but concerning proportion of homes showing disproportionately higher methane concentrations.

• If indoor-outdoor differences were tested statistically (e.g., paired t-tests, Wilcoxon).

In the Data Analysis Section, on lines 188-191 (tracked changes revision) we stated "We used the Wilcoxon Sign-Rank test for paired samples of outdoor and indoor air for no gas homes and a paired t-test was used to compare mean indoor and outdoor [CH4] concentrations in the gas group."

• How measurements that change over time (like cooking times and heating cycles) were dealt with.
It would be beneficial to clearly indicate whether the observed differences are statistically significant and how the variability among households was addressed.

In our large sample of homes, we did not assess or attempt to manage how visit times corresponded to heating and cooking cycles. To the extent that incomplete combustion from gas stoves or heaters added methane to the ambient air, these would have added to elevated methane concentrations detected, and increased the overall variability of results when mixed with sampling of homes when gas stoves or heaters were not operating. Despite this uncertainty, our statistical results are clear that homes served with gas have statistically significantly higher methane compared to outdoor air and homes without gas service.

P-values indicating statistical significance in observations are listed in Tables 1 and 2.

On lines 263-268 (tracked changes revision) we state: "There did not appear to be a relationship between home attributes or geography and elevated [CH4] in homes served by gas. For example, the 10 homes showing the highest elevation of [CH4] relative to outdoors were located in nine different municipalities and in both states. Of these top 10 homes, all contained basements and first floors, eight had second floors, and three had third floors, and all ten were found in single

family homes. There was no relationship between indoor [CH4] and either home age (P > 0.1) or square footage (P > 0.1).”

3. There are a number of things that can affect indoor methane levels, like how much air flows through the house, whether there are gas leaks, how often appliances are used, and how tight the house is. However, these factors are not controlled in a systematic way. Please let us know if any leak detection or verification was done.

• The behaviour of the people living there or the time of year affected the measurements.
• Conditions outside (like wind and temperature) may have affected the gradients between inside and outside. A brief discussion of sensitivity would bolster the conclusions.

Generally speaking, all of the factors mentioned above are critical in determining indoor methane concentrations. However, we took a “big data” approach to measure from a large sample of households at the expense of being able to conduct detailed studies of ventilation, insulation, and the types and ages of appliances in each home, or to monitor wind and temperature conditions during our sampling. Having performed the more detailed study as discussed in Nicholas et al. (2023), we are well aware of the difference between the detailed flux approach we took in that study versus this study with large sample size but simple and incomplete documentation of all important factors. We have discussed these substantial limitations of our study on lines 399-407 and hope that the limited scope of our study spurs further research that can fill the important areas of detail that were beyond the scope of this study.

As for leak detection or verification, as stated on lines 255-262 (tracked changes revision), “We had the opportunity to conduct brief searches for suspected methane sources for elevated [CH4] in 137 (78%) of the 175 homes with gas service. Of these homes, we positively identified leak sources in 124 homes, or in 91% of homes checked. Leak sources were found all along the distribution system within homes: pipe connection (63), appliance malfunction or incomplete burning (53), shutoff valves (13), migration (3) and corrosion (1). Eighteen (18) of the leaks came from gas company pipes. Beyond the statistical association of higher [CH4] in homes with gas, this provides further evidence linking elevated [CH4] in homes to incomplete combustion, and leaks from gas pipes, connections, and appliances.”

4. The conversation brings up important issues about the effects of climate change and the quality of indoor air, but the implications could be made clearer. Things to think about:
• How this study compares to other recent studies on gas or methane leaks in buildings.

• The importance of the measured concentrations in relation to safety thresholds, even if they are much lower than acute hazards.

• Possible policies or ways to reduce the problem (for example, appliance maintenance or ventilation advice). The manuscript would be better if it had a little more context.

We believe that we addressed this important broader context issues in our introduction and discussion sections, citing recent highly relevant papers like Rowland et al. (2024) and discussing the relationship between our study and other related studies and the unique contribution our study makes. We note that we mention “climate” five times in the introduction,

and have explicit sections in the discussion on "Safety and Methane Emissions", and "Air Quality and Health".

As for policies and ways to reduce the problem, we have made an important specific policy recommendation on lines 319-321 (tracked changes revision), to inspect leak-prone service lines annually, a higher frequency than is in current practice. Beyond that, we think that our finding that homes without gas service overwhelmingly show lower indoor methane is an important demonstration of benefits of household heating electrification.

Minor comments
1. Please make sure that all units (ppm, ppb) are used the same way in the text and figures.

All uses of "ppm" are consistent throughout the text and figures (abbreviated as ppm and lower case). We have not use "ppb" in the text or figures.

2. Legends and axis labels on figures would be easier to understand, especially on graphs with more than one panel.

Legends and axis labels on all figures have been added.

3. A table that lists the characteristics of the household (like having a gas stove or furnace, the age of the home, etc.) would help readers understand the differences.

We feel that we provided basic household information concisely in the introductory paragraph of the results section (lines 197-204).

4. Some of the sentences in the introduction sound the same; making them shorter will help the flow.

We have reviewed the introduction and shortened sentences to reduce redundancy. Reviewers can view these shortened sentences in the track changes version of the manuscript. Thank you for the helpful suggestion.

5. Please check for small grammar mistakes and tense inconsistencies.

We have completed a grammar and spell check on the revised manuscript.
