# Supplementary material for: Correction: Indoor methane consistently above outdoor levels in homes with natural gas service
Source: PLoS One. 2026 Aug 26;21(8):e0357112. doi: 10.1371/journal.pone.0357112 (PMC13514018; doi:10.1371/journal.pone.0357112)
Supplement: Supplementary file 1 [file pone.0357112.s001.docx]

**Appendix 1.**

*Instrument calibration check*

We tested the analyzer prior to the beginning of the survey, on February 29, 2024; during a midpoint of the survey, on April 23, 2024, and near the conclusion of the survey on June 19, 2024, against nominal 0.0 ppm; 2.0 ppm and 10 ppm test gasses in ultrapure air. The test gas tanks (Scott-Marrin, Riverside, CA USA) were certified to contain < 0.01 ppm; 2.072 ppm; and 10.32 ppm [CH_4_] respectively (+/- 1% NIST). Test gasses were supplied to the analyzer using Tedlar bags filled with test gasses and connected to the analyzer inlet during normal operation. Tedlar bags of test gas supply were maintained until the graphical data display indicated the analyzer [CH_4_] reading stabilized near the nominal value. Table 3 shows the match between analyzer values of [CH_4_] and test gas values. These results demonstrate that our analyzer was working properly and with adequate precision for the study.

**Table 3.** Analyzer calibration checks.

| **Date** | **[CH**_4_**] = < 0.01 ppm** | **[CH**_4_**] = 2.072 ppm** | **[CH**_4_**] = 10.32 ppm** |
| --- | --- | --- | --- |
| 2/29/2024 | 0.011 | 2.042 | 10.21 |
| 4/23/2024 | 0.008 | 2.046 | 10.06 |
| 6/19/2024 | 0.027 | 2.019 | 10.07 |

**Table 4.** Values (in ppm) for Figure 1.

|  | **Basement Minus Outdoor**  **No Gas** | **Basement Minus Outdoor**  **Gas** | **First Floor Minus Outdoor**  **No Gas** | **First Floor Minus Outdoor**  **Gas** | **Second Floor Minus Outdoor**  **No Gas** | **Second Floor Minus Outdoor**  **Gas** | **Third Floor Minus Outdoor**  **No Gas** | **Third Floor Minus Outdoor**  **Gas** |
| --- | --- | --- | --- | --- | --- | --- | --- | --- |
| Min | -0.20 | -0.10 | -0.07 | -0.05 | -0.09 | -0.06 | -0.06 | 0.01 |
| Quart. 1 | -0.04 | 0.31 | -0.01 | 0.26 | -0.01 | 0.27 | -0.06 | 0.21 |
| Median | -0.01 | 0.80 | 0.03 | 0.60 | 0.03 | 0.60 | -0.02 | 0.49 |
| Quart. 3 | 0.03 | 1.98 | 0.18 | 1.36 | 0.19 | 1.44 | 0.11 | 1.10 |
| Max | 0.71 | 38.20 | 0.71 | 20.78 | 0.46 | 17.43 | 0.34 | 17.17 |
| Mean | 0.03 | 1.92 | 0.10 | 1.32 | 0.09 | 1.29 | 0.06 | 1.29 |
| Standard Deviation | 0.18 | 3.80 | 0.19 | 2.31 | 0.17 | 2.18 | 0.19 | 2.77 |
| IQR  (Q3-Q1) | 0.06 | 1.67 | 0.19 | 1.10 | 0.21 | 1.17 | 0.17 | 0.97 |
| Lower Bound | -0.13 | -2.19 | -0.30 | -1.39 | -0.32 | -1.49 | -0.31 | -1.24 |
| Upper Bound | 0.12 | 4.48 | 0.47 | 3.01 | 0.50 | 3.20 | 0.36 | 2.63 |
